# Neoadjuvant hepatic arterial infusion of oxaliplatin, fluorouracil, and leucovorin for resectable single large hepatocellular carcinoma

**DOI:** 10.1097/JS9.0000000000002437

**Published:** 2025-05-12

**Authors:** Zili Hu, Min Deng, Yizhen Fu, Zhongguo Zhou, Huanwei Chen, Minshan Chen, Yaojun Zhang

**Affiliations:** a Department of Liver Surgery, Sun Yat-sen University Cancer Center, Guangzhou, People’s Republic of China; b Collaborative Innovation Center for Cancer Medicine, State Key Laboratory of Oncology in South China, Sun Yat-Sen University Cancer Center, Guangzhou, People’s Republic of China; c Guangdong Provincial Clinical Research Center for Cancer, Sun Yat-Sen University Cancer Center, Guangzhou, People’s Republic of China; d Department of General Surgery, The Seventh Affiliated Hospital of Sun Yat-sen University, Shenzhen, People’s Republic of China; e Department of Hepatic Surgery, The Affiliated Foshan Hospital of Sun Yat-Sen University, Foshan, People’s Republic of China

**Keywords:** hepatic arterial infusion chemotherapy, hepatocellular carcinoma, hepatoectomy, neoadjuvant therapy, single large tumor

## Abstract

**Background and aims:**

Patients diagnosed with single large hepatocellular carcinoma (HCC) often face a daunting prognosis and pose a treatment challenge. In this study, we aimed to evaluate the effectiveness of neoadjuvant hepatic arterial infusion chemotherapy (HAIC) with oxaliplatin, fluorouracil, and leucovorin (FOLFOX) in patients with single large HCC.

**Methods:**

397 patients with resectable single, ≥7 cm HCC from three centers in China between January 2016 and December 2021 were included, 268 patients underwent hepatectomy alone and 129 patients underwent neoadjuvant HAIC. The log-rank test was used to compare the overall survival (OS) and disease-free survival (DFS) by intension-to-treat analysis between the two groups.

**Results:**

The 1-, 3-, and 5-year OS rates were 83.3%, 62.9%, and 53.8% in the surgery alone group, and 97.5%, 80.7%, and 64.7% in the neoadjuvant HAIC group. The 1-, 3-, and 5-year DFS rates were 48.8%, 32.5%, and 26.2% in the surgery alone group, and 71.5%, 61.7%, and 59.5% in the neoadjuvant HAIC group. The neoadjuvant HAIC group showed significantly longer OS (hazard ratio [HR], 0.506; 95% confidence interval [CI], 0.347–0.734; *P* < 0.001) and DFS (HR, 0.466; 95% CI, 0.357–0.609; *P* < 0.001) than the surgery alone group. There was no HAIC-related death in the neoadjuvant HAIC group.

**Conclusions:**

Neoadjuvant FOLFOX-HAIC significantly improved the OS and DFS with acceptable toxicities in HCC patients with resectable single, ≥7 cm tumor.

## Graphical abstract

**Figure:**
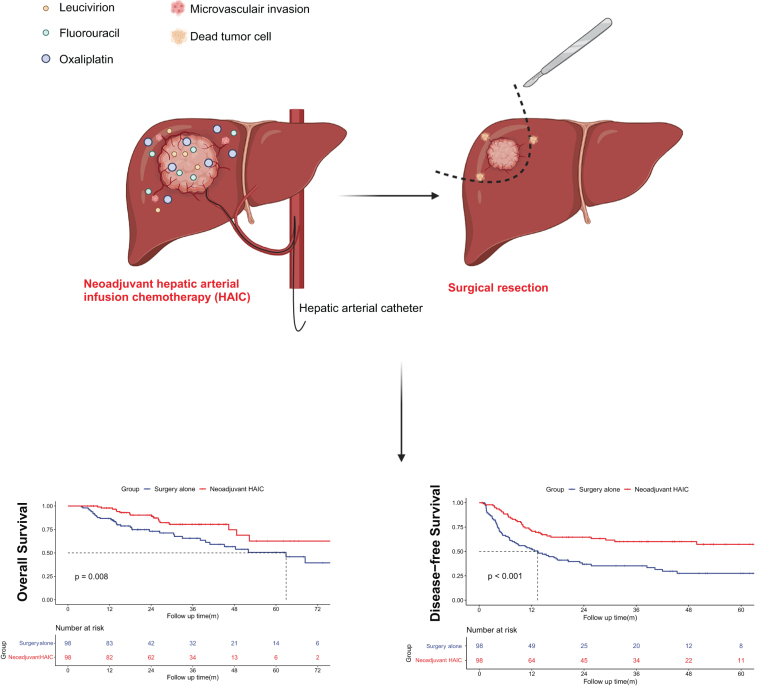

## Introduction

Hepatocellular carcinoma (HCC) is the third leading cause of cancer-related death globally^[^1^]^. Due to its elusive symptoms, HCC is frequently diagnosed at an advanced stage^[^2^]^. Many individuals remain undetected until their tumors have reached to a large size or even huge size^[^3^]^. Patients with single large HCCs generally face a poor prognosis and pose significant treatment challenges^[^4^]^. According to various guidance^[^5-8^]^, surgical resection is regarded as the preferred option for single large HCCs without macrovascular invasion and with preserved liver function. However, R0 resection of single large HCCs is challenging and is accompanied by high rates of postoperative complications and recurrence^[^9,10^]^. Consequently, there is an urgent need for effective neoadjuvant therapy for single large HCCs.

**HIGHLIGHTS**

- The shrinking of tumor after neoadjuvant HAIC made more remnant liver available.
- Neoadjuvant HAIC eliminated the potential small metastasis in the liver.
- There was no significant change in liver functional reserve after neoadjuvant HAIC. Neoadjuvant HAIC improved the OS and DFS in resectable single, ≥7 cm HCC.

Previously, transarterial chemoembolization (TACE) was investigated as the neoadjuvant therapy for resectable large HCC in a randomized phase III trial^[^11^]^. Unfortunately, the study reported that patients who received neoadjuvant TACE had similar overall survival (OS) and disease-free survival (DFS) after hepatectomy compared to those who underwent hepatectomy alone. This outcome may be attributed to the challenges associated with achieving complete embolization for large HCCs due to abundant extrahepatic collateral arteries^[^12^]^, and plenty of embolization particles are needed to embolize large HCC leading to deterioration of hepatic functional reserve^[^13,14^]^.

Compared to TACE, hepatic arterial infusion chemotherapy (HAIC), offering sustained high concentrations of chemotherapy agents to the tumors without the need for embolization, may be a promising neoadjuvant therapy for single large HCCs. Several studies have confirmed the efficacy of FOLFOX-HAIC in HCC patients with large tumor burden^[^15-18^]^. In China, FOLFOX-HAIC has been recommended for HCC patients with tumor sizes larger than 7 cm or with portal vein tumor thrombus^[^19^]^.Moreover, a previous randomized phase III trial reported that postoperative adjuvant FOLFOX-HAIC significantly improved the DFS benefits in HCC patients with microvascular invasion (MVI)^[^20^]^. Large HCC is often accompanied by the presence of MVI. Therefore, this study aims to evaluate the effect of neoadjuvant HAIC on the survival of patients with single, ≥7 cm HCC.

## Patients and methods

### Patients

In this multicenter retrospective cohort study, we enrolled patients with single, ≥7 cm HCCs from January 2016 to December 2021 at three centers in China. The study was approved by the ethics committee of the three centers [B2022-238-01] and was carried out in accordance with the guidelines outlined in the Declaration of Helsinki. Informed consent was waived because this study was retrospective. The work has been reported in line with the STROCSS criteria^[^21^]^

Inclusion criteria for patients were as follows: (1) hepatocellular carcinoma (HCC) confirmed by contrast-enhanced triphasic computed tomography (CT) and/or magnetic resonance imaging (MRI), demonstrating both early arterial enhancement and delayed washout, in accordance with the American Association for the Study of Liver Diseases (AASLD) Practice Guideline for HCC Management^[^22^]^; (2) age between 18 and 75 years; (3) primary HCC with single, ≥7 cm tumor; (4) performance status score of 0 or 1; (5) Child–Pugh A; (6) indocyanine green retention rate at 15 minutes (ICG-R15) of less than 20%; (7) sufficient residual functional liver volume after operation: residual liver volume in patients without cirrhosis accounted for ≥35% of standard liver volume and residual liver volume in patients with cirrhosis accounted for ≥45% of standard liver volume. Patients who met the following criteria were excluded: (1) history of other malignancies; (2) previous antitumor treatment for HCC; (3) presence of macrovascular invasion or distant metastasis; (4) recurrent HCC. There were 397 patients suitable for surgical resection included in the end. Patients with tumor in close contacted with the main vessel, unclear tumor boundary, and a high risk of post-hepatoectomy liver failure were recommended to receive neoadjuvant HAIC. Taking physician’s recommendation, physical conditions, and patient’s wishes into consideration, finally, 268 patients underwent resection alone and 129 patients underwent neoadjuvant HAIC.

### HAIC procedure

The HAIC procedure was carried out following established protocols from prior studies^[^15-18^]^. Initially, a femoral artery puncture was performed, followed by catheterization and positioning of the catheter within the hepatic artery using digital subtraction angiography (DSA). The catheter was then connected to an infusion pump in the ward, and the following chemotherapy regimen was administered: oxaliplatin (130 mg/m^2^) infused from hour 0 to 2 on day 1; leucovorin (400 mg/m^2^) from hour 2 to 3 on day 1; fluorouracil (400 mg/m^2^) as a bolus at hour 3; and a continuous infusion of fluorouracil (2400 mg/m^2^) over 46 hours from days 1 to 3. Upon completion of the regimen, the catheter and sheath were promptly removed. For each HAIC cycle, typically repeated every 3 weeks, the puncture and catheterization process was repeated. Tumor response was assessed approximately every 6 weeks using MRI and/or CT scans, with evaluations conducted by two experienced radiologists based on the modified Response Evaluation Criteria in Solid Tumors (mRECIST)^[^23^]^. Following the assessment of treatment response, a multidisciplinary team discussion was held to determine the suitability of surgical resection. This decision took into account the physician’s recommendation, the patient’s overall physical condition, and their personal preferences.

The patients were assessed before each HAIC procedure according to the National Cancer Institute Common Terminology Criteria for Adverse Events version 4.0 (CTCAE v4.0). HAIC was delayed until recovery if the neutrophil count was less than 1.2 × 10^9^/L, the platelet (PLT) count was less than 60 × 10^9^ platelets/L, the total bilirubin (TBIL) exceeded 30 mmol/L, the albumin (ALB) was less than 3.0 mg/dL, the aspartate transaminase (AST) and alanine transaminase (ALT) exceeded 5 times the upper limit of the normal range, or the serum creatinine was up to 1.5 times the institutional upper limit of normal. In cases of grade 3 major organ drug-related toxicity, the dose of 5-fluorouracil was reduced to 300 mg/m^2^ as a bolus and 1800 mg/m^2^ as a continuous infusion. The dose of oxaliplatin was reduced to 85 mg/m^2^ in cases of grade 3 or 4 neutropenia or thrombocytopenia, any other grade 3 major organ drug-related toxicity, or abdominal pain.

### Surgical resection procedure

The procedure was carried out by highly skilled surgeons, each with over a decade of surgical expertise. To accurately define the tumor margins and detect any potential nodules, intraoperative ultrasonography was employed before the liver ligaments were separated. The liver tissue was then precisely dissected using a harmonic scalpel. Once hemostasis was thoroughly achieved, the surgical site and abdominal cavity were flushed with sterile water. A peritoneal drain was inserted in cases where blood loss surpassed 200 ml or there was a possibility of bile leakage.

### Follow-up

During neoadjuvant HAIC, follow-up was performed about every 6 weeks. After Surgical resection, follow-up was performed in the first months and every 2–3 months thereafter. Following surgical resection, patients were monitored closely during the initial months of post-operation, with subsequent follow-ups scheduled every 2–3 months. These follow-up assessments comprised laboratory analyses, including serum alpha-fetoprotein (AFP) levels, liver function tests, and routine blood work, as well as imaging studies such as contrast-enhanced MRI and/or CT scans. The study’s primary endpoint was OS, defined as the period from HCC diagnosis to either death or the last follow-up. The secondary endpoint was DFS, calculated from the date of diagnosis with HCC to disease progression.

### Statistical analysis

To minimize selection bias and address potential confounders between the two groups, a propensity score matching (PSM) analysis was conducted. Propensity scores were derived using a multivariable logistic regression model, by incorporating the following variables: age, cirrhosis, tumor diameter, PLT, prothrombin time (PT), AFP, ALT, AST, albumin–bilirubin (ALBI) grade. Patients were matched in a 1:1 ratio with a caliper width of 0.2, employing the nearest neighbor matching method via the R package “MatchIt” (Supplementary Figure 1, available at: http://links.lww.com/JS9/E118). Categorical variables were compared between the two groups using Fisher’s exact test and Pearson’s chi-square test. OS and DFS were evaluated using Kaplan-Meier curves, with statistical significance assessed via the log-rank test. Risk factors influencing OS and DFS were analyzed using univariable and multivariable Cox proportional hazards models. Variables with a *P* value <0.05 in the univariable analysis were included in the multivariable model. All statistical analyses were conducted using SAS (version 26.0, SAS Institute, Cary, NC, USA) and R statistical software (version 3.6.4, https://www.R-project.org/).

## Results

### Baseline characteristics of patients

**Figure 1.**
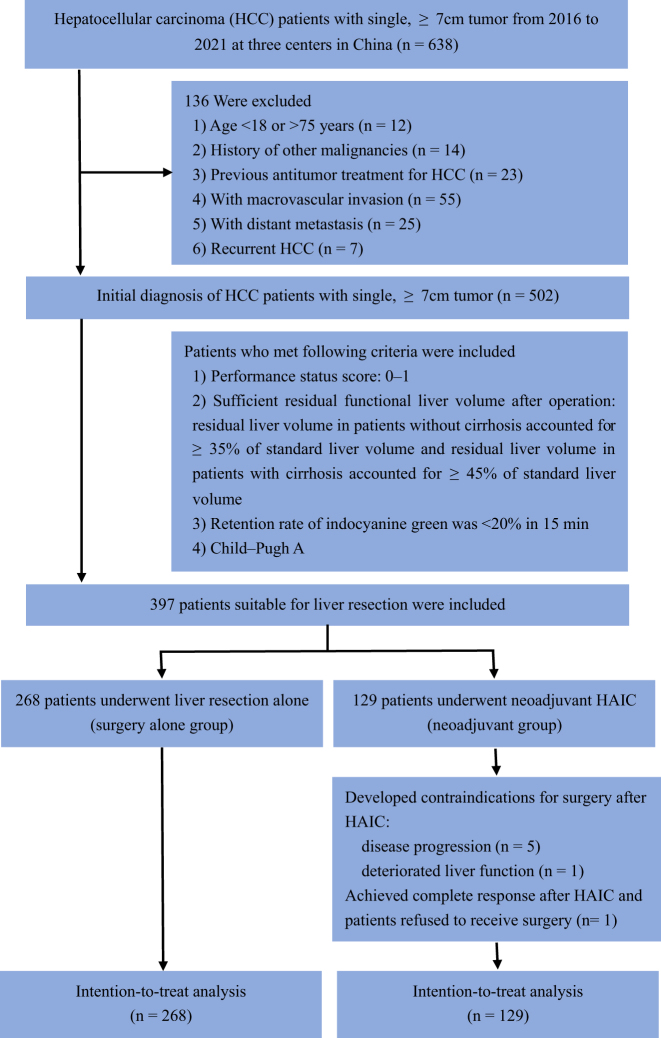
Flow diagram for the patient selection process.

From January 2016 to December 2021, 397 HCC patients with single, ≥7 cm tumors who were eligible for liver resection were included in this study (Fig. 1). Among these patients, 268 underwent liver resection alone and 129 received neoadjuvant HAIC. In the neoadjuvant HAIC group, six patients developed contraindications to surgery after HAIC and one patient declined surgery due to complete response after HAIC, and they underwent nonsurgical treatment (targeted therapy or combination of targeted therapy and immunotherapy, n = 5; supportive therapy, n = 1; persistent follow-up, n = 1). The average number of times of HAIC procedure was 2.6. Through PSM (ratio 1:1), we created two new cohorts consisting of 98 patients each in the surgery alone group and the neoadjuvant HAIC group, respectively, and the two groups were well-matched as indicated by a standardized mean difference of less than 10% for all baseline variables (Supplementary Figure 2, available at: http://links.lww.com/JS9/E119).

In the intension-to-treat (ITT) cohort, the median duration of follow-up was 41.8 months (95% confidence interval [CI], 37.5–46.1). Compared to the surgery alone group, the neoadjuvant HAIC group had a higher proportion of patients with age ≥60 years (31.8% vs. 21.6%), cirrhosis (41.1% vs. 29.1%), AST ≥40 U/L (70.5% vs. 57.8%), ALBI grade 1 (74.4% vs. 34.3%). After PSM, there were no significant differences in baseline characteristics between the two groups. The baseline characteristics were collected at the time of initial diagnosis of HCC, and detailed information is summarized in Table 1. For pathologic characteristics, the neoadjuvant HAIC group had fewer patients with microvascular invasion (MVI) in both ITT cohort (20.5% vs. 49.3%) and PSM cohort(22.1% vs. 44.9%) compared to the surgery alone cohort.

**Table 1:** Baseline and pathologic characteristics of patients in the intension-to-treat (ITT) cohort and the propensity matching (PSM) cohort

	ITT cohort				PSM cohort		
	Surgery alone group (n = 268)	Neoadjuvant HAIC group (n = 129)	*P* value		Surgery alone group (n = 98)	Neoadjuvant HAIC group (n = 98)	*P* value
Baseline characteristics							
Age, years			0.039				1.000
<60	210(78.4)	88(68.2)			78(79.6)	78(79.6)	
≥60	58(21.6)	41(31.8)			20(20.4)	20(20.4)	
Gender			0.824				0.862
Male	216(80.6)	102(79.1)			76(77.6)	78(79.6)	
Female	52(19.4)	27(20.9)			22(22.4)	20(20.4)	
HBV infection			0.865				0.657
Absence	20(7.5)	11(8.5)			13(13.3)	10(10.2)	
Presence	248(92.5)	118(91.5)			85(86.7)	88(89.8)	
Cirrhosis			0.024				1.000
Absence	190(70.9)	76(58.9)			59(60.2)	58(59.2)	
Presence	78(29.1)	53(41.1)			39(39.8)	40(40.8)	
Tumor diameter, cm			0.369				0.198
<10	131(48.9)	70(54.3)			57(58.2)	47(48.0)	
≥10	137(51.1)	59(45.7)			41(41.8)	51(52.0)	
Platelet, x10^9^/L			0.327				1.000
>100	257(95.9)	120(93.0)			91(92.9)	91(92.9)	
≤100	11(4.1)	9(7.0)			7(7.1)	7(7.1)	
PT, s			0.721				1.000
<13.5	242(90.3)	115(89.1)			90(91.8)	90(91.8)	
≥13.5	26(9.7)	14(10.9)			8(8.2)	8(8.2)	
ALT, U/L			0.163				1.000
<40	140(52.2)	57(44.2)			48(49.0)	47(48.0)	
≥40	128(47.8)	72(55.8)			50(51.0)	51(52.0)	
AST, U/L			0.020				0.882
<40	113(42.2)	38(29.5)			37(37.8)	35(35.7)	
≥40	155(57.8)	91(70.5)			61(62.2)	63(64.3)	
AFP, ng/mL			<0.001				0.886
<400	166(61.9)	55(42.6)			50(51.0)	48(49.0)	
≥400	102(38.1)	74(57.4)			48(49.0)	50(51.0)	
ALBI			<0.001				1.000
Grade 1	92(34.3)	96(74.4)			66(67.3)	66(67.3)	
Grade 2	175(65.3)	33(25.6)			32(32.7)	32(32.7)	
Grade 3	1(0.4)	0(0)			0(0)	0(0)	
**Pathologic characteristics**							
MVI			<0.001				0.001
Absence	136(50.7)	97(79.5)			54(55.1)	74(77.9)	
Presence	132(49.3)	25(20.5)			44(44.9)	21(22.1)	
Tumor differentiation			0.926				0.395
I/II	142(53.0)	57(51.8)			48(49.0)	49(56.3)	
III/IV	126(47.0)	53(48.2)			50(51.0)	38(43.7)	
Surgical margin, cm			0.591				0.623
≤1	191(71.3)	96 (74.4)			71 (72.4)	75 (76.5)	
>1	77 (28.7)	33 (25.6)			27 (27.6)	23 (23.5)	

Categorical variables are described as frequencies and percentages. AFP, alpha fetoprotein; ALBI, albumin–bilirubin; ALT, alanine aminotransferase; AST, aspartate aminotransferase; HBV: hepatitis B virus; MVI, microvascular invasion; PT, prothrombin time.

### Overall survival analysis between surgery alone and neoadjuvant HAIC groups

**Figure 2.**
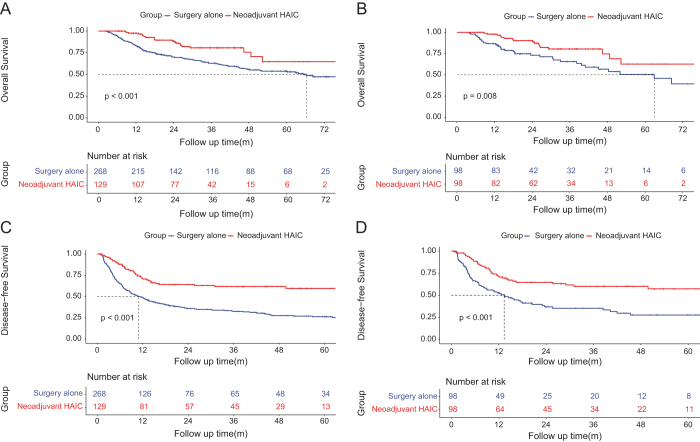
Kaplan–Meier curves of overall survival between the surgery alone group and the neoadjuvant HAIC group in the intension-to-treated cohort (A) and the matched cohort (B); Kaplan–Meier curves of disease-free survival between the surgery alone group and the neoadjuvant HAIC group in the intension-to-treated cohort (C) and the matched cohort (D).

In the ITT cohort, the median OS was 66.3 (95% CI, 59.2–83.3) months in the surgery alone group and unreached in the neoadjuvant HAIC group. The 1-, 3-, and 5-year OS rates were 83.3%, 62.9%, and 53.8% in the surgery alone group, and 97.5%, 80.7%, and 64.7% in the neoadjuvant HAIC group. In the PSM cohort, the median OS was 62.9 (95% CI, 42.5–83.3) months in the surgery alone group and unreached in the neoadjuvant HAIC group. The 1-, 3-, and 5-year OS rates were 86.7%, 65.6%, and 50.5% in the surgery alone group, and 97.8%, 80.4%, and 62.6% in the neoadjuvant HAIC group. The neoadjuvant HAIC group exhibited significantly longer OS than the surgery alone group both in the ITT cohort (hazard ratio [HR], 0.506; 95% CI, 0.347–0.734; *P* < 0.001; Fig. 2A) and in the PSM cohort (HR, 0.480; 95% CI, 0.279–0.828; *P* = 0.008; Fig. 2B).

Univariate analyses of OS were presented in Supplementary Table 1 (available at: http://links.lww.com/JS9/E121) multivariable analyses revealed that PT > 13.5s (HR, 1.972; 95% CI, 1.154–3.219; *P* = 0.012), AFP > 400 ng/mL (HR, 1.789; 95% CI, 1.265–2.531; *P* = 0.001), microvascular invasion (MVI; HR, 2.316; 95% CI, 1.611–3.329; *P* < 0.001) and neoadjuvant HAIC (HR, 0.505; 95% CI, 0.309–0.825; *P* = 0.006) were independent risk factors associated with OS (Table 2).

**Table 2:** Multivariate analysis of prognostic factors on overall survival and disease-free survival

Variable	Comparison	Overall survival		Disease-free survival	
		HR (95% CI)	*P* value	HR (95% CI)	*P* value
**ALBI**				1.37(1.036–1.811)	0.027
**PT, s**	<13.5 vs. ≥13.5	1.927(1.154–3.219)	0.012		
**AFP, ng/mL**	<400 vs. ≥400	1.789(1.265–2.531)	0.001	1.494(1.146–1.948)	0.003
**MVI**	Absence vs. presence	2.316(1.611–3.329)	<0.001	1.887(1.444–2.465)	<0.001
**Types of treatment**	Surgery alone vs. neoadjuvant HAIC	0.505(0.309–0.825)	0.006	0.577(0.404–0.824)	0.002

CI, confidence interval; HR, hazard ratio; MVI, microvascular invasion; PT, prothrombin time.

### Disease-free survival between surgery alone and neoadjuvant HAIC groups

In the ITT cohort, the median DFS was 10.9 (95% CI, 8.0–13.8) months in the surgery alone group and unreached in the neoadjuvant HAIC group. The 1-, 3-, and 5-year DFS rates were 48.8%, 32.5%, and 26.2% in the surgery alone group, and 71.5%, 61.7%, and 59.5% in the neoadjuvant HAIC group. In the PSM cohort, the median DFS was 13.4 (95% CI, 7.2–19.5) months in the surgery alone group and unreached in the neoadjuvant HAIC group. The 1-, 3-, and 5-year DFS rates were 52.8%, 35.3%, and 27.6% in the surgery alone group, and 71.8%, 60.1%, and 57.3% in the neoadjuvant HAIC group. The neoadjuvant HAIC group exhibited significantly longer DFS than the surgery alone group both in the ITT cohort (HR, 0.466; 95% CI, 0.357–0.609; *P* < 0.001; Fig. 2C) and in the PSM cohort (HR, 0.440; 95% CI, 0.296–0.656; *P* < 0.001; Fig. 2D).

Univariate analyses of DFS were presented in Supplementary Table 1 (available at: http://links.lww.com/JS9/E121) multivariable analyses revealed that ALBI grades 2 and 3 (HR, 1.37; 95% CI, 1.036–1.811; *P* = 0.027), AFP > 400 ng/mL (HR, 1.949; 95% CI, 1.146–1.948; *P* = 0.003), microvascular invasion (HR, 1.887; 95% CI, 1.444–2.465; *P* < 0.001) and neoadjuvant HAIC (HR, 0.557; 95% CI, 0.404–0.824; *P* = 0.002) were independent risk factors associated with DFS (Table 2).

### HAIC-related outcomes

During neoadjuvant HAIC, 13(10.1%) patients had complete response (CR), 62(48.1%) patients had partial response (PR), 49 (38.0) patients had stable disease (SD), and 5(3.9) patients had progressive disease (PD) according to mRECIST^[^23^]^. The overall response rate (ORR) was 58.2%. The level of AFP before surgery showed a significant decrease compared to the level before HAIC (Supplementary Figure 3A and B, available at: http://links.lww.com/JS9/E120). During the HAIC procedure, the most common HAIC-related AEs were abdominal pain (37.2%), elevated AST (34.1%), and fever (25.6%) (Supplementary Table 2, available at: http://links.lww.com/JS9/E122). The nonsteroidal anti-inflammatory drugs and anisodamine were routinely implemented to prevent abdominal pain. The serum level of AST increased significantly immediately following the completion of HAIC treatment but returned to normal within one week.

**Figure 3.**
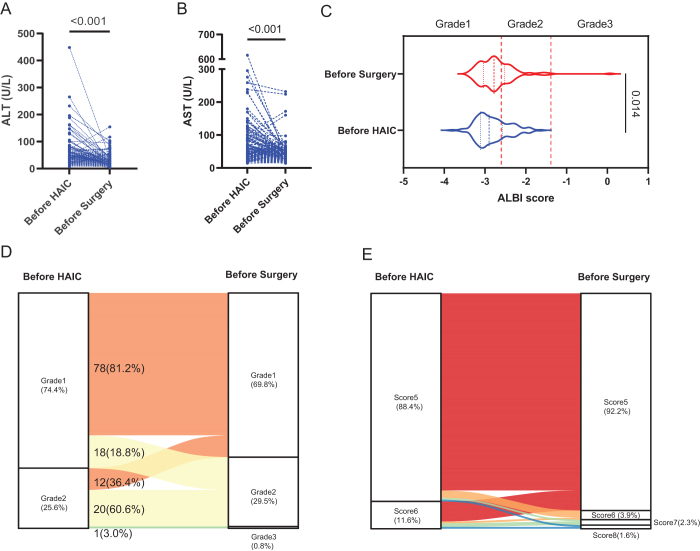
The level of ALT (A) and AST (B) before HAIC and before surgery; violin plot of ALBI score and grade before HAIC and before surgery (C); Sankey diagram of ALBI grade change after neoadjuvant HAIC(D); Sankey diagram of Child–Pugh score change after neoadjuvant HAIC (E).

Interestingly, the level of ALT (56.0 ± 46.1 vs. 31.2 ± 24.1 U/L, *P* < 0.001; Fig. 3A) and AST (73.9 ± 71.8 vs. 43.4 ± 33.2 U/L, *P* < 0.001; Fig. 3B) showed a significant decrease after the entire course of neoadjuvant HAIC. The ALBI score increased after the neoadjuvant HAIC (−2.82 ± 0.40 vs. −2.72 ± 0.45, *P* = 0.014; Fig. 3C) but there was no significant difference in ALBI grade between before HAIC and before surgery (grade 1, 74.4% vs. 69.8%, *P* = 0.462; Fig. 3D). Five patients experienced a change from Child–Pugh class A (score 5,6) to class B (score 7,8) after neoadjuvant HAIC (Fig. 3E). Among them, one patient developed deteriorated liver function, leading to contraindications for surgery.

### Operative outcomes

Compared with the surgery alone group, the neoadjuvant HAIC group had a higher proportion of patients with operative blood loss ≤400 ml (71.6% vs. 54.0%, *P* = 0.018) and shorter hospital stays (11.0 vs. 14.3 days, *P* < 0.001). Regarding postoperative complications, the surgery alone group had a higher incidence of hepatic insufficiency (21.4% vs. 8.4%, *P* = 0.020), while the neoadjuvant HAIC group had a higher incidence of bile leakage (9.5% vs. 1.0%, *P* = 0.008). There was no significant difference in operation time (184.2 vs. 172.7 mins, *P* = 0.241) between the two groups. Detailed operative outcomes are described in Table 3.

**Table 3:** Operative outcomes between the surgery alone group and the neoadjuvant HAIC group in the matched cohort

	Surgery alone group	Neoadjuvant HAIC group	*P* value
	(n = 98)	(n = 95)	
Hospital stays(days)	14.3 (10.0, 18.6)	11.0(8.1, 13.9)	<0.001
Operation time(mins)	184.2 (121.8, 246.6)	172.7(120.3, 225.1)	0.241
Operative blood loss (N, %)			0.018
≤400 ml	53 (54.0)	68 (71.6)	
>400 ml	45 (46.0)	27 (28.4)	
Postoperative complications (N, %)			
Absent	60 (61.2)	67(70.5)	0.226
Hepatic insufficiency a	21 (21.4)	8 (8.4)	0.020
Bile leakage b	1(1.0)	9 (9.5)	0.008
Thorax/ peritoneal effusion	4 (4.1)	8 (8.4)	0.342
Pulmonary/ peritoneal infection	4 (4.1)	2 (2.1)	0.429
Postoperative hemorrhage	8(8.2)	9 (9.5)	0.946
Intestinal obstruction	1 (1.0)	0 (0)	0.324
Others	2 (2.0)	0 (0)	0.162
90-day mortality (N, %)	0 (0)	0 (0)	/

Categorical variables are described as frequencies and percentages. Continuous variables are described as mean ± standard deviation (SD).

^a^ Diagnosis based on 50–50 criteria.

^b^ Diagnosis based on International Study Group of Liver Surgery Standards (ISGLS).

## Discussion

The efficacy of systemic chemotherapy with the FOLFOX regimen in treating advanced HCC has been confirmed by the results of EACH study^[^24^]^. Recently, several randomized trials have demonstrated the survival benefit of FOLFOX-HAIC in HCC patients with large tumor burden^[^15,16^]^. Li’s randomized study substantiated the superior efficacy of FOLFOX-HAIC over TACE in patients with ≥7 cm, unresectable HCC without vascular invasion or extrahepatic metastasis^[^15^]^. Lyu’s randomized study demonstrated better survival outcomes of FOLFOX-HAIC compared to sorafenib in locally advanced HCC^[^16^]^. In China, FOLFOX-HAIC has been recommended for HCC patients with tumor sizes larger than 7 cm or with portal vein tumor thrombus^[^19^]^. In this study, we demonstrated that neoadjuvant FOLFOX-HAIC provided favorable survival benefits for HCC patients with resectable single, ≥7 cm tumors, and our results suggested that FOLFOX-HAIC was well-tolerated and exhibited acceptable safety.

The single large HCC typically has a rich blood supply from the hepatic artery, via which chemotherapy agents can be sustainably pumped to the tumor and sustain stable local high concentrations in the tumor for more than 24 hours. Moreover, removing embolization preserves the patency of the supply artery for repeated delivery of chemotherapy agents. Those above may explain the high ORR of FOLFOX-HAIC in single large HCCs. Neoadjuvant HAIC-induced tumor volume shrinkage provides more remnant liver volume, which might lead to reduced post-surgical complications and mortality during the perioperative period. Indeed, we observed a higher incidence of hepatic insufficiency after surgery in the surgery alone group compared to the neoadjuvant HAIC group (Table 3). This may be one of the reasons why neoadjuvant HAIC benefits patients with single large tumors. However, we also observed an increased incidence of bile leakage in the neoadjuvant HAIC group, which might be attributed to small bile duct injuries caused by the chemotherapy drugs during neoadjuvant HAIC.

In the current study, most patients benefited from DFS within the first 2 years. Micrometastasis in the residual liver was the main cause of early HCC recurrence after hepatectomy, which is a high-risk outcome associated with MVI^[^25,26^]^. The sustainable infusion of chemotherapy agents has the potential to eliminate the small metastasis in the blood circulation and liver parenchyma. Furthermore, Li’s study has proven that adjuvant FOLFOX-HAIC after surgery significantly reduces recurrence in HCC patients with MVI^[^20^]^. Large HCCs are often accompanied by the presence of MVI. In this study, 49.3% of patients had MVI in the control group, whereas the presence of MVI was only 20.5% in the neoadjuvant HAIC group (Table 1). The significant decrease in MVI may be attributed to neoadjuvant HAIC, thereby providing another reason for the beneficial effects of neoadjuvant HAIC in patients with single large tumors.

The impact of neoadjuvant HAIC on liver function should be noted. Generally, the level of ALT and AST significantly increased on the day immediately following the completion of HAIC treatment but returned to normal within one week. Interestingly, the level of ALT and AST before surgery showed a significant decrease compared to the level before HAIC. The reason might be that the baseline levels of ALT and AST exceeded the reference range at the initial diagnosis of HCC because most patients in this study were infected with HBV and they did not receive any treatment before. After positive anti-virus and liver-protecting therapy, the levels of ALT and AST decreased to normal despite intervention with neoadjuvant chemotherapy. The ALBI score before surgery increased after the neoadjuvant HAIC (Fig. 3C), but there was no significant difference in ALBI grade between before HAIC and before surgery (Fig. 3D), indicating no significant change in liver functional reserve after neoadjuvant HAIC. However, it should be noted that five of 129 patients transitioned from Child–Pugh class A (score 5,6) to class B (score 7,8) after neoadjuvant HAIC (Fig. 3E). Among them, one patient developed deteriorated liver function, leading to contraindications for surgery. Overall, neoadjuvant HAIC was well-tolerated.

The study has several limitations. First, as it is a retrospective study, selection bias is inevitable. Although patients were enrolled from three centers in this study, which may reduce the selection bias, further studies with prospective evaluations are needed to confirm the findings. Second, since all patients enrolled in this study are Chinese and most of them are infected with HBV, the value of neoadjuvant HAIC in patients with single large HCC with different ethnic groups and hepatitis backgrounds requires further investigation. Finally, each cycle of HAC requires patients to stay in bed for longer than 48 hours, which may affect the patients’ treatment compliance. The trial protocol needs further optimization.

In conclusion, this study suggested that neoadjuvant FOLFOX-HAIC significantly improved the OS and DFS with acceptable toxicities in HCC patients with resectable single, ≥7 cm tumors.

## Data Availability

None.
